# A case of inflammatory pseudotumor of the lung presenting as polymyalgia rheumatica

**DOI:** 10.1002/ccr3.2172

**Published:** 2019-06-26

**Authors:** Erin L. Tompkins, Candice E.‐P. Middlebrook, Christopher L. Tracy

**Affiliations:** ^1^ Womack Army Medical Center Fort Bragg North Carolina

**Keywords:** acute medicine, lung, polymyalgia rheumatica, pseudotumor

## Abstract

A common inflammatory condition, the investigation and diagnosis of polymyalgia rheumatica should be within the scope of a general physician's repertoire. While there is limited evidence to confirm the existence of polymyalgia rheumatica as a distinct paraneoplastic syndrome (*Reumatismo*. 2018;**70**(1):23), a broadened differential should be utilized prior to diagnosis of this disease.

## 1 INTRODUCTION

Polymyalgia rheumatica (PMR) is a relatively common inflammatory condition that generally occurs in patients 50 years and older, characterized by aching and morning stiffness specifically in the shoulder and pelvic girdles.^1^ Though inflammatory pseudotumors have been reported in other rheumatologic conditions,^2^, ^3^, ^4^, ^5^ we present a case of an inflammatory pseudotumor of the lung in a patient with clinically appearing PMR.

## 2 CASE REPORT

A 50‐year‐old Caucasian female with hypothyroidism presented to an outpatient clinic for evaluation after 1 year of fatigue, unplanned weight loss of 10 pounds, proximal hip, and shoulder girdle pain that had not improved with physical therapy and daily NSAID use. A clinical diagnosis of polymyalgia rheumatica was suspected based on findings of proximal hip and shoulder girdle bursitis and the presence of elevated inflammatory markers in the absence of specific antibodies or peripheral synovitis. She had near immediate response to 20 mg daily prednisone with subsequent normalization of her inflammatory markers.

During subsequent rheumatologic review of her medical history, she endorsed a chronic intermittently productive cough with an associated foul taste and smell. Due to this, additional laboratory work and chest imaging were performed (Table 1: Patient laboratory values).

**Table 1:** Laboratory values

Variable	Result (Reference range)
White blood cell count, ×1000/μL	8.9 (3.2‐10.8)
Neutrophils, %	76.8 (44.5‐78.4%)
Lymphocytes, %	15.6 (13.7‐43.7%)
Eosinophils, %	1.4 (0.2‐5.9%)
Basophils, %	0.4 (0.1‐1.2%)
Monocytes, %	5.8 (3.7‐11.4%)
Platelet count, ×1000/μL	298 (125‐352)
Blood urea nitrogen, mg/dL	13.5 (6‐20)
Creatinine, mg/dL	0.93 (0.5‐0.9)
Aspartate aminotransferase, U/L	14 (0‐32)
Alanine aminotransferase, U/L	10 (0‐33)
Total bilirubin, mg/dL	0.3 (0‐1.2)
Albumin, g/dL	4.4 (3.5‐5.2)
Protein, g/dL	7.6 (6.2‐8.3)
International normalized ratio	1.0
Thyrotropin	2.4 μIU/mL
Erythrocyte sedimentation rate, mm/hr	55
Creatinine kinase	97 U/L
Rheumatoid factor/cyclic citrullinated peptide IgG/IgA	Negative
Quantiferon tuberculosis evaluation	Negative
Infectious hepatitis serologic panel	Negative for hepatitis C, non‐reactive hepatitis B surface Ag, non‐reactive hepatitis B core Ab

She denied sick contacts, had no infectious exposures in travel and no previous exposures to tuberculosis. She was a lifelong non‐smoker.

**Figure 1:**
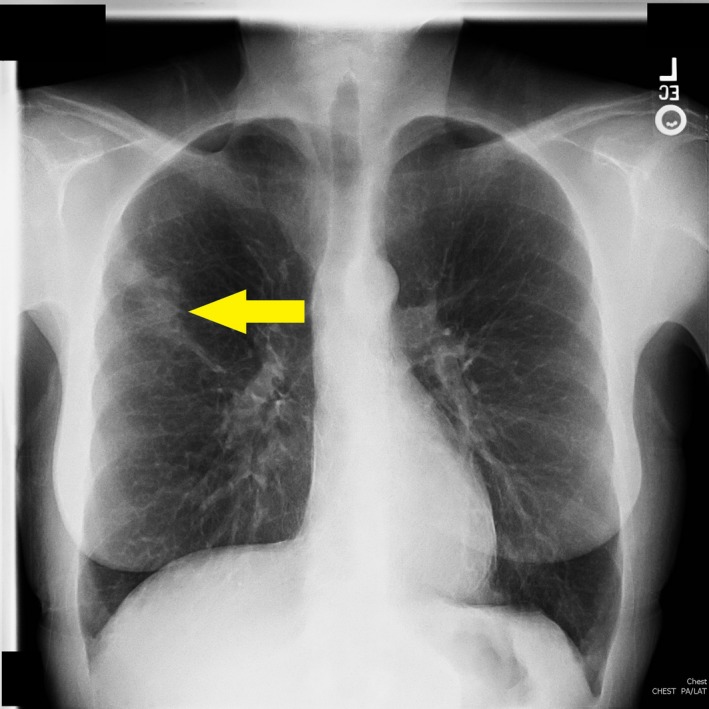
Inflammatory pseudotumor plain film

**Figure 2:**
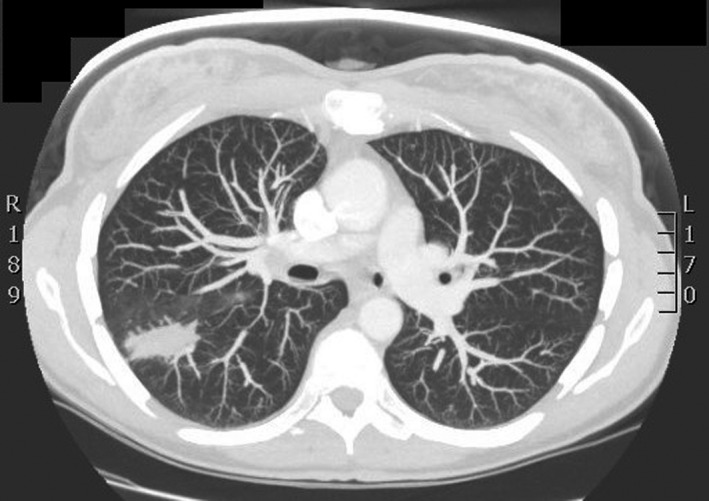
Inflammatory pseudotumor contrasted CT chest

An initial chest X‐ray revealed an incidental right upper lobe lung mass (Figure 1). A malignant process was suspected after a CT scan of the chest revealed a 5.0 cm right pulmonary irregular, spiculated cavitary mass (Figure 2) that subsequently showed a PET active right mediastinal lymph node.

The patient elected to undergo a right lung robot‐assisted video‐assisted thoracoscopic surgery (VATS) wedge resection after non‐diagnostic trans‐bronchial biopsies. The gross specimen was described as a nodule that measured 1.1 cm at widest dimension and crossed both upper and lower lobes of the lung. Staining of the lesion was negative for IgG and IgG‐4 immunopositive plasma cells. Other features of IgG4‐related disease such as sclerosis or obliterative vascular patterns were also not identified. Pleura over the mass was red‐tan and roughened but did not appear adherent or puckered. Cut surface of the mass was tan‐gray with a stellate appearance and had areas of calcification, identified by pathologic analysis as an inflammatory pseudotumor.

The patient recovered from surgery and tolerated a very slow taper of the low dose corticosteroid treatment without recurrence of her presenting symptoms of hip and shoulder girdle pain.

## 3 DISCUSSION

Inflammatory pseudotumors are rare lesions best described as well‐circumscribed, non‐encapsulated masses of unregulated cellular growth. These lesions are generally not uniform and may contain elements of inflammation, hemorrhagic features, calcification and rarely, cavitation.^6^, ^7^ Inflammatory pseudotumors have been reported in a variety of organ systems to include the central nervous system, liver, spleen, salivary glands, soft tissue and skin as well as in the lung.^6^, ^7^, ^8^ Etiology of these lesions remains unclear; however, case reports suggest that inflammatory pseudotumors represent an exaggerated immunologic response^7^, ^9^, ^10^ and possibly impaired T‐cell function.^11^ Many believe that these lesions could be triggered by the body's reaction to a viral or foreign antigen reaction such as to minor trauma, mycobacteria, Epstein‐Barr Virus, Actinomyces, Nocardia, mycoplasma, or Herpes Simplex Virus.^6^, ^7^, ^9^, ^10^, ^11^ They have also been associated with malignancies.^3^, ^6^

When evaluated microscopically, it is typically discovered that inflammatory pseudotumors consist of a variety of cellular elements, making histological diagnosis difficult. They are generally composed of fibrous tissue, inflammatory cells, and characteristically large numbers of plasma cells.^4^, ^8^ The plasma cells are polyclonal in nature with immunoglobulin G predominance, specifically IgG‐4.^4^, ^6^, ^8^, ^12^ In the lung, these lesions may be parenchymal or endobronchial. Considered cellularly benign, they rarely invade the mediastinum, but can impinge on nearby structures.^13^ These lesions also have the potential to recur, which is thought to be due primarily to incomplete resection.^6^

Inflammatory pseudotumors of the lung are relatively rare, especially in adults, and are thought to represent <1% of all reported lung lesions.^6^, ^7^ They are difficult to diagnose clinically, as patients are often asymptomatic, due to the development of these lesions in an indolent manner.^9^ There have not been specific findings documented on physical or laboratory examination to suggest the presence of an inflammatory pseudotumor, but patients often have reported weight loss, fever, and fatigue with or without a cough.^6^, ^8^, ^14^, ^15^ Several case reports state that inflammatory pseudotumors of the lung have been discovered incidentally on chest radiograph^3^ as with the case we describe.

Although these lesions of the lung are rare, they can cause significant morbidity in patients due to their size^16^ and also present a diagnostic challenge due to their ability to mimic malignancy on imaging.^15^, ^17^ Many of these lesions are PET positive^8^, ^13^ and can even be spiculated on plain film or CT, raising concern for a malignant process.^7^ There is some debate as to whether inflammatory pseudotumors are considered reactive lesions or more closely resemble true neoplasms. Reports of pediatric patients describe lesions with cytogenic characteristics of tumors, whereas adult patients tend to have lesions with more inflammatory, non‐neoplastic, and fibrotic content.^15^, ^17^ Pseudotumors do not always have predictable behavior and can be quite aggressive.^6^, ^9^, ^18^ They have been reported to invade locally, have distant metastases,^15^ and in some cases have recurred after resection.^19^

Though there are case reports of complete resolution of inflammatory pseudotumors of the lung with pharmacotherapy,^8^ complete surgical resection remains the most frequently recommended course of treatment.^3^, ^6^, ^20^ Resection is performed both to exclude malignancy and to achieve cure as local expansion can cause morbidity.^10^, ^13^, ^15^, ^16^ If the lesion is unable to be completely resected, cannot be resected due to location or size, or in the case of multifocal disease, reported treatments have included radiotherapy, chemotherapy, and corticosteroids.^3^, ^13^ Prognosis after surgical resection of these lesions is excellent.^14^ Complete resection, when possible, has been associated with excellent survival^16^ and remains the best method known to prevent recurrence.^6^, ^16^, ^21^

## 4 CONCLUSION

Here we present a case of an inflammatory pseudotumor of the lung in a patient with a clinical PMR presentation or possibly a paraneoplastic presentation secondary to this inflammatory lesion. Polymyalgia rheumatica is a relatively common inflammatory condition that generally occurs in patients 50 years and older, and would explain our patient's other clinical symptoms. It is classically characterized by proximal joint stiffness specifically in the shoulder and pelvic girdles. There have been rare reports of inflammatory pseudotumors within the cervicothoracic spine^3^ in patients with established polymyalgia; however, none specifically reported in the lung of a patient with PMR only. Inflammatory pseudotumors have been described in patients with other rheumatologic conditions, such as dermatomyositis and granulomatosis with polyangiitis.^4^, ^5^ Lesions are reported both in a multifocal pattern, as well as solitary findings described in the brain, esophagus, stomach, liver, spleen, lymph nodes, salivary glands, testes, breast, soft tissues, and skin.^4^, ^5^, ^6^, ^11^ Although rare, inflammatory pseudotumors of the lung should be considered as part of the differential diagnosis when a pulmonary lesion is discovered in a patient with an unexplained inflammatory condition. We urge clinicians to remember the potential of PMR to be a paraneoplastic disease, as there is an established connection between malignancy and rheumatologic conditions.^1^, ^2^ Additional research is needed to discover the relationship, if one exists, between this type of pulmonary lesion and polymyalgia rheumatica.

## DISCLAIMER

The views expressed herein are those of the authors and do not reflect the official policy or position of the U.S. Army Medical Department, Department of the Army, Department of Defense, or the U.S. Government.

## CONFLICT OF INTEREST

The authors have no conflict of interests to disclose.

## AUTHOR CONTRIBUTION

ET: completed the background research, drafted, and edited the manuscript. ET, CEPM, CT: edited the manuscript and approved the final manuscript.

## INFORMED CONSENT

Informed consent was obtained from the patient for educational use of the below mentioned data and no personal patient information has been disclosed.
